# *APOE* ɛ4 allele and *TOMM40*‐*APOC1* variants jointly contribute to survival to older ages

**DOI:** 10.1111/acel.13730

**Published:** 2022-11-03

**Authors:** Alexander M. Kulminski, Ethan Jain‐Washburn, Ian Philipp, Liang He, Yury Loika, Elena Loiko, Olivia Bagley, Svetlana Ukraintseva, Anatoliy Yashin, Konstantin Arbeev, Eric Stallard, Mary F. Feitosa, Nicole Schupf, Kaare Christensen, Irina Culminskaya

**Affiliations:** ^1^ Biodemography of Aging Research Unit, Social Science Research Institute Duke University Durham North Carolina USA; ^2^ Division of Statistical Genomics, Department of Genetics Washington University School of Medicine St Louis Missouri USA; ^3^ Gertrude H. Sergievsky Center Columbia University Irving Medical Center New York New York USA; ^4^ Unit of Epidemiology, Biostatistics and Biodemography, Department of Public Health Southern Denmark University Odense Denmark

**Keywords:** aging, Alzheimer's disease, apolipoprotein E polymorphism, haplotypes, linkage disequilibrium

## Abstract

Age‐related diseases characteristic of post‐reproductive life, aging, and life span are the examples of polygenic non‐Mendelian traits with intricate genetic architectures. Polygenicity of these traits implies that multiple variants can impact their risks independently or jointly as combinations of specific variants. Here, we examined chances to live to older ages, 85 years and older, for carriers of compound genotypes comprised of combinations of genotypes of rs429358 (*APOE* ɛ4 encoding polymorphism), rs2075650 (*TOMM40*), and rs12721046 (*APOC1*) polymorphisms using data from four human studies. The choice of these polymorphisms was motivated by our prior results showing that the ɛ4 carriers having minor alleles of the other two polymorphisms were at exceptionally high risk of Alzheimer's disease (AD), compared with non‐carriers of the minor alleles. Consistent with our prior findings for AD, we show here that the adverse effect of the ɛ4 allele on survival to older ages is significantly higher in carriers of minor alleles of rs2075650 and/or rs12721046 polymorphisms compared with their non‐carriers. The exclusion of AD cases made this effect stronger. Our results provide compelling evidence that AD does not mediate the associations of the same compound genotypes with chances to survive until older ages, indicating the existence of genetically heterogeneous mechanisms. The survival chances can be mainly associated with lipid‐ and immunity‐related mechanisms, whereas the AD risk, can be driven by the AD‐biomarker‐related mechanism, among others. Targeting heterogeneous polygenic profiles of individuals at high risks of complex traits is promising for the translation of genetic discoveries to health care.

## Graphical abstract

The APOE ɛ4 allele and minor alleles of rs2075650 *TOMM40* and rs12721046 *APOC1* variants confer a higher risk of Alzheimer's disease compared with the ɛ4 allele and no minor alleles of these two polymorphisms. Here, we show that the same relationship characterizes chances to live 85 years and older. Nevertheless, we show that Alzheimer's disease does not mediate the associations of the same compound genotypes with chances to survive to 85+ years, indicating the existence of genetically heterogeneous mechanisms.

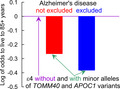

**Abbreviations**
AD: Alzheimer’s disease
APOE: Apolipoprotein E
TOMM40: Translocase of Outer Mitochondrial Membrane 40
APOC1: Apolipoprotein C1
SNP: single nucleotide polymorphism
LD: linkage disequilibrium
LLFS: Long Life Family Study
FHS: Framingham Heart Study
CHS: Cardiovascular Health Study, UKB, UK Biobank
HRC: Haplotype Reference Consortium
CARDIA: Coronary Artery Risk Development in Young Adults
NIA: National Institute on Aging
Aβ: amyloid‐β
PET: Positron Emission Tomography
dbGaP: database of Genotypes and Phenotypes
MA: minor allele
SE: standard error

## 1 BACKGROUND

Diseases and related traits associated with older age, the process of aging itself (senescence), longevity, and chances to live to older ages are examples of complex, non‐Mendelian phenotypes (herein referred to as age‐related traits). Heritability estimates suggest that these traits can have a genetic component. For example, studies of Swedish twins estimated the heritability of the liability of a devastating age‐related disease such as late‐onset Alzheimer's disease (AD) at 45% for women and 58% for men (Gatz et al., 2006). Heritability of life span is smaller, and it is unlikely above 23% (van den Berg et al., 2017).

The complexity of the age‐related traits suggests, however, that the contributions of genetic variants may be intricate, with at least two major entangled factors. The first is a complex and redundant structure of the macromolecular organization in humans, evolutionarily adapted to specific environments in the past (Barabasi & Oltvai, 2004; Goh et al., 2007; Morange, 2000). The second is at best the indirect role of evolution in mechanisms of age‐related traits (Nesse et al., 2012). Accordingly, the genetic contributions to age‐related traits could manifest in various forms including, for example, the impacts of causal variants, structural diversity of the genome, intricate genetic architectures of complex traits, combinations of risk alleles, haplotypes, and combinations of genotypes (called compound genotypes) (Eichler et al., 2010; Franceschi et al., 2018; Gibson, 2012; Kulminski et al., 2021; Rogaev et al., 1995; Wainberg et al., 2020; Zhou et al., 2019). Dissecting the genetic complexity is a challenging task as exemplified by the well‐studied apolipoprotein E (*APOE*) ε2/ε3/ε4 polymorphism; despite decades of *APOE* research, its role in age‐related traits is still unclear (Belloy et al., 2019; Genin et al., 2011).

Because non‐Mendelian age‐related traits are polygenic, they can be impacted by multiple variants. A common approach to handling the impacts of multiple variants is to aggregate the effects of the risk alleles from multiple variants, such as single nucleotide polymorphisms (SNPs), in a polygenic risk score. Another approach considers the effects of combinations of specific variants in a person in the forms of haplotypes or compound genotypes. The difference between these two approaches is that the former disregards specific features of genetic variants (i.e., considering that any combination of any risk alleles confers the risk of the trait), whereas the latter highlights the impact of combinations of certain alleles. These differences result in different views on the potential linkage disequilibrium (LD) between SNPs whereby the former approach considers LD as a complicating factor whereas the latter does not.

Given the polygenicity of age‐related traits, prioritization of promising variants for the association analyses of multiple SNPs with a targeted trait using either approach is beneficial. Recently, we used an approach to prioritize promising SNPs based on the analysis of the differences in LD structures between AD‐affected and unaffected subjects (Kulminski, Philipp, et al., 2020; Kulminski, Shu, et al., 2020). As a result, we found that carriers of the *APOE* ε4 allele (encoded by rs429358 minor allele) who also carry minor alleles of rs2075650 (*TOMM40*) and rs12721046 (*APOC1*) SNPs were at remarkably high excess risk of AD compared with carriers of the major alleles of these two SNPs. Here, we extend these analyses by examining the relationship between the identified AD‐predisposing compound genotypes comprising variants from the rs429358, rs2075650, and rs12721046 SNPs and the chances of living to older ages, 85 years and older—using data from four major studies: the Long Life Family Study (LLFS), the Framingham Heart Study (FHS), the Cardiovascular Health Study (CHS), and the UK Biobank (UKB) cohort.

## 2 METHODS

### 2.1 Study cohorts

We obtained the data for the present paper from the LLFS (Wojczynski et al. 2022), three FHS cohorts (Cupples et al., 2009), the CHS (Fried et al., 1991), and the UKB (Sudlow et al., 2015) (Table 1). The FHS and CHS are community‐based studies, the UKB is a population‐based study, and the LLFS is targeted at participants from families showing exceptional familial longevity and their spouses at three field centers in the U.S. and one in Denmark. These datasets were selected because they targeted younger and older populations, which include a relatively large number of the *APOE* ε4 carriers and were not enriched for this allele due to enrichment of AD‐affected subjects. All analyses were performed using individuals of European ancestry. See also the Appendix S1.

**TABLE 1:** Basic characteristics of the genotyped participants of European ancestry in the selected studies.

Sample	All				85+ years		<65 years	
	Age, mean (SD), years	Age range	*N*	Men, %	*N*	Men, %	*N*	Men, %
LLFS	79.0 (14.1)	26.9–111.0	4692	45.0	1623	44.9	778	39.3
FHS	68.4 (16.0)	22.9–109.8	8867	46.0	1597	36.0	3849	48.1
CHS	83.4 (5.4)	66.2–103.9	4376	43.7	1550	44.5	0	0
UKB	65.4 (8.0)	40.8–83.1	459,225	45.7	0	0	200,575	44.5

*Note*: Age was defined at the end of follow‐up or death.

Abbreviations: CHS, Cardiovascular Health Study; FHS, Framingham Heart Study; LLFS, Long Life Family Study; SD, standard deviation; UKB, UK Biobank dataset.

### 2.2 Main outcomes

We employed a case/control design with cases defined as subjects who exceeded the highest average life expectancies in developed countries—with a representative cut‐off at 85 years—and controls defined as relatively young subjects, that is, younger than 65 years at the time of last contact (Table 1). Although some controls will live beyond age 85, the 20‐year gap between the minimum age of cases and the maximum age of controls is sufficient to reliably conduct the analyses in the present paper given the high survival selection due to the more than 14‐fold increase in death rates between ages 55–64 and 85+ (Murphy et al., 2021). This gap did, however, introduce one complication to our analysis. Specifically, because the CHS did not include subjects younger than age 65 years and the UKB did not have subjects older than 85 years, we used a pooled sample of CHS and UKB (CHS&UKB) subjects to define the case/control outcomes.

### 2.3 Genotypes

Compound genotypes were constructed using three SNPs, which contributed to the exceptionally high risk of AD (Kulminski et al., 2022), rs429358 (*APOE*, T/c; upper/lower case denotes here major/minor allele; allele “c” encodes the ε4 allele), rs2075650 (*TOMM40*, A/g), and rs12721046 (*APOC1*, G/a). To increase the sample size, we imputed (Michigan Imputation Server, HRC panel) missing genotypes for some subjects in each study and retained genotypes with high imputation quality (*r* ^*2*^ > 0.8). To select samples with no carriers of the *APOE* ε2 allele, we also included rs7412 (C/t) SNP, whose minor allele encodes the ε2 allele.

Although for the three bi‐allelic SNPs there are 27 compound genotypes, that is, 3^3^, the vast majority of them were rare (Table S1). Our goal was to examine associations of three common individual compound genotypes—TT/AA/GG (complete major allele homozygote), Tc/Ag/Ga (complete heterozygote), and Tc/AA/GG—and five aggregated compound genotypes identified in our prior study of AD (Kulminski et al., 2022) (Table 2). To streamline notations, we also defined compound genotypes based on the counts of minor alleles in an SNP. For example, the Tc/AA/GG genotype having one minor allele in rs429358_Tc is denoted as “100.” Five aggregated compound genotypes include combinations of individual compound genotypes. For example, genotype denoted as 1XY aggregates rs429358_Tc (“1”) and all genotypes of rs2075650 (“X”) and rs12721046 (“Y”), except major allele homozygotes of both SNPs, rs2075650_AA and rs12721046_GG (“00”), because it is included in the “100” genotype.

**TABLE 2:** Meta‐analysis of the estimates of the log odds *β* of living to 85 years and older (85+ years).

Genotype	MA coding	*N*_cases_	*N*_controls_	*β*	*SE*	*p*‐Value	Direction	*I* ^2^, %	*p*‐Het
TT/AA/GG	000	3028	125,334	Refer					
Aggregated	0XY	518	20,684	0.084	0.061	1.72E‐01	+++	0	8.45E‐1
Tc/AA/GG	100	161	9206	−0.206	0.099	3.70E‐02	− −−	0	3.94E‐1
Tc/Ag/Ga	111	473	37,083	−0.429	0.059	3.25E‐13	− − −	0	4.87E‐1
Aggregated	1XY	581	44,561	−0.394	0.054	3.94E‐13	− − −	0	7.39E‐1
Aggregated	100+200	165	9362	−0.196	0.097	4.36E‐02	− − −	8.30	3.36E‐1
Aggregated	111+222	492	39,766	−0.448	0.058	1.01E‐14	− − −	0	5.42E‐1
Aggregated	1XY+2XY	612	49,283	−0.429	0.053	6.48E‐16	− − −	0	9.15E‐1

*Note*: Meta‐analysis aggregated the results from CHS&UKB, FHS, and LLFS. Symbols in the “**Direction**” column show directions of the effects in these samples, in that order. Column “**Genotype**” shows actual compound genotypes constructed from SNPs ordered as rs429358, rs2075650, and rs12721046, and aggregated compound genotypes. “**MA coding:**” individual (000, 100, and 111) and aggregated (0XY, 1XY, 100+200, 111+222, and 1XY+2XY) compound genotypes are coded based on counts of minor alleles 0, 1, or 2 in each SNP in the same order. Symbols “X” and “Y” denote aggregated compound genotypes, which include more than two compound genotypes. Symbols “X” and “Y” take values of 0, 1, or 2 but not simultaneously 0. The negative direction implies that the chances of living to 85+ years are smaller for carriers of genotypes shown in the “**MA coding**” column compared to the reference 000 genotype. Columns ***N***_**cases**_ and ***N***_**controls**_ show the number of cases and controls, respectively. Cases and controls were defined as subjects who were 85 years and older (85+ years) and younger than 65 years at the end of follow‐up or right censoring. Study‐specific results are given in Table S2.

Abbreviations: *I* ^2^, heterogeneity coefficient; *p*‐het, heterogeneity *p*‐value; *SE*, standard error.

### 2.4 Statistical analysis

We evaluated the chances of living to older ages for carriers of the selected compound genotypes constructed from the rs429358, rs2075650, and rs12721046 SNPs in the entire samples and the samples with AD‐affected subjects excluded. We used the affection status defined by the neurologic exam criteria (McKhann et al., 1984, 2011) in FHS, the International Classification of Disease codes in CHS and UKB, and self or proxy reports in LLFS. We employed the base R function *glm* for logistic regression. The models were adjusted for sex and field centers (LLFS). The analysis of the FHS and LLFS studies, which included participants from families, was performed using the *glmer* function from the *lme4* R package fitting a generalized linear mixed‐effects model with a random effect for the intercept to adjust for familial clustering. The analyses were not adjusted for principal components because such an adjustment may have an adverse effect on survival‐ and longevity‐related studies due to mortality selection (Yashin et al., 2014). Meta‐analysis was performed using a fixed‐effects model with inverse‐variance weighting. We used *p* < 0.05 as the significance level for all tests. We examined the consistency of the directions of the effects in different studies that “is widely regarded as replication” (Marigorta et al., 2018).

### 2.5 Supplementary analysis

We also conducted supplementary case/control analyses (Tables S4–S5) in which we redefined the cases to be subjects who lived beyond age 85, but without AD, and the controls to be subjects younger than 65 years, also without AD, at the time of the last contact. Consistent with the low incidence of AD below age 65, the supplementary controls included 99.94% of the primary controls. In contrast, the supplementary cases included only 77.1% of the primary cases. The exclusion of AD cases did not change the average ages in the case and control groups.

### 2.6 Sensitivity analysis

We also performed two types of sensitivity analyses in the samples with AD‐affected subjects excluded. First, we used the cut‐off for cases at slightly older (87 years) and younger (83 years) ages and the same cut‐off for control, 65 years. We did not examine the cut‐off at 90 years and older because the samples of cases become small in all studies. Second, we used the original cut‐off at 85 years for cases and 65 years for controls, but the Coronary Artery Risk Development in Young Adults (CARDIA) study (Hughes et al., 1987) as controls (mean age = 40.5 and SD = 3.8 years) instead of UKB.

## 3 RESULTS

Table 2 and Table S2 show that the effects *β* (logarithm of odds ratio) characterizing the chances of living to 85 years and older (85+) are smaller (i.e., the *β*s are more negative) for carriers of all compound genotypes bearing the ɛ4 allele, compared with carriers of the complete major allele homozygote 000. The chances of living to 85+ years for non‐carriers of the ɛ4 allele who have at least one minor allele of rs2075650 or rs12721046 (Table 2, 0XY) do not differ significantly from those for carriers of the 000 genotype. The effects *β* tend to be consistently smaller for carriers of the ɛ4 allele who have at least one minor allele of rs2075650 or rs12721046 compared with those who do not have minor alleles of these two SNPs. For example, the log odds for carriers of the 111 heterozygote (*β* = −0.429) are more than 2‐fold smaller than those for carriers of the 100 genotype (*β* = −0.206).

**FIGURE 1:**
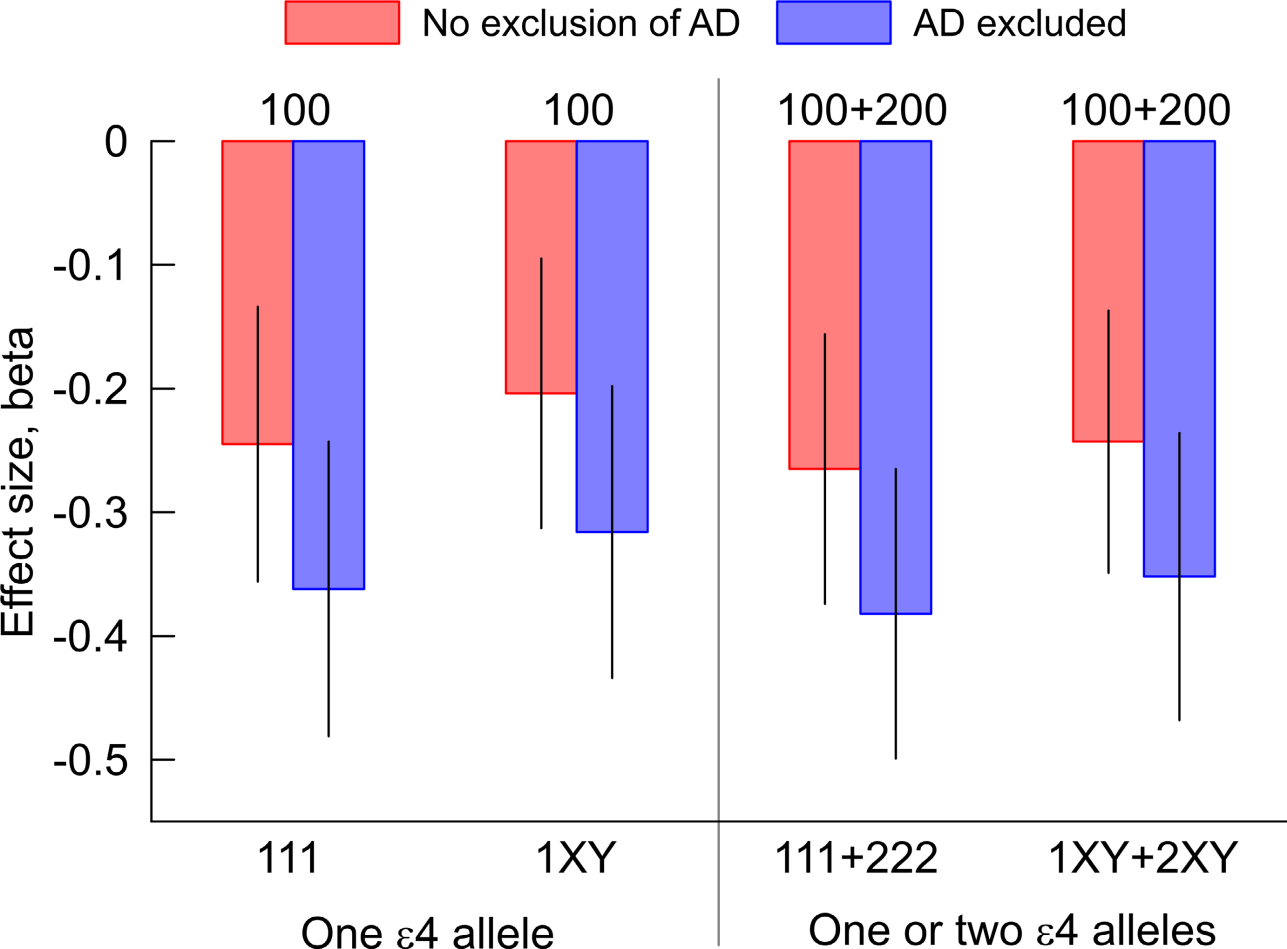
Reductions of the log odds *β* of living to 85 years and older. The negative direction implies that the chances are smaller for carriers of genotypes shown below the *x*‐axis than those shown at the top of the figure. Individual (100 and 111) and aggregated (1XY, 100+200, 111+222, and 1XY+2XY) compound genotypes are coded based on counts of minor alleles 0, 1, or 2 in each SNP ordered as rs429358, rs2075650, and rs12721046. Symbols “X” and “Y” denote aggregated compound genotypes, which include more than two compound genotypes. Symbols “X” and “Y” take values of 0, 1, or 2 but not simultaneously 0. Red: no exclusion of AD, blue: AD‐affected subjects were excluded. Vertical lines show standard errors. Numerical estimates are in given Tables S3 and S4.

Next, we contrasted the chances of living to 85+ years for the ɛ4 carriers with at least one minor allele of rs2075650 or rs12721046 to those for the ɛ4 carriers without minor alleles of these two SNPs. We found that people having the ɛ4 allele and minor alleles of rs2075650 and rs12721046 had consistently smaller chances of living to 85+ years in each study and in the meta‐analysis than subjects with major alleles of both SNPs (Figure 1 and Table S3). The meta‐analysis showed that the one individual (111) and three aggregated (1XY, 111+222, and 1XY+2XY) compound genotypes with at least one minor allele of rs2075650 and rs12721046 either significantly (111, 111+222, and 1XY+2XY) decreased the chances of living to 85+ years or marginally significantly (1XY) decreased these chances.

Given that the compound genotypes comprising rs429358, rs2075650, and rs12721046 contributed to an exceptionally high risk of AD (Kulminski et al., 2022), we examined whether the exclusion of AD‐affected subjects impacted the estimates of *β* for ɛ4 carriers who have at least one minor allele of rs2075650 or rs12721046 compared with those with major alleles of both SNPs (Figure 1 and Table S4). The study‐specific analysis and meta‐analysis showed that the chances of living to 85+ years without AD were consistently and substantially smaller (i.e., the *β*s were uniformly more negative and their statistical significances were uniformly larger in each analysis) than the results in Table S3 which were computed without consideration of AD status. Carrying a complete minor allele heterozygote (111) or homozygote (222) had the strongest impact on the chances of living to 85+ years without AD, *β* = −0.382, *p* = 1.10 × 10^−3^. The differences in the effects in the samples with AD excluded (Table S4) and without consideration of the AD status (Table S3) did not attain statistical significance, as evidenced by overlapping standard errors in Figure 1.

The studied compound genotypes can include a small number of subjects carrying the ɛ2 allele in the ɛ2/ɛ4 heterozygous genotype. Because the ɛ2 allele can be favorable for surviving to older ages, we verified that the exclusion of the ɛ2 carriers had trivial impacts on these estimates (Table S5).

Our sensitivity analysis using 83 or 87 years (instead of 85 years) as cut‐off defining cases at older ages showed that carriers of the ɛ4 allele that have minor alleles of rs2075650 and/or rs12721046 had consistently smaller chances to live to those ages compared with those who did not carry these two minor alleles (Table S6). We also observed that the log odds became even smaller for living to slightly older ages, 87+ years, e.g., *β* = −0.504 (*p* = 3.54 × 10^−4^) for carriers of a complete minor allele heterozygote (111) or homozygote (222). The sensitivity analysis replacing UKB with CARDIA cohort did not alter the estimates (Table S7).

## 4 DISCUSSION

Various studies show that carriers of the ɛ4 allele live shorter lives and have higher risks of death (Deelen et al., 2019; Drenos & Kirkwood, 2010; Kulminski et al., 2014; Nygaard et al., 2014; Raichlen & Alexander, 2014; Sebastiani et al., 2019; Wolters et al., 2019; Yashin et al., 2018). Some studies implicate the ɛ4 allele as the only risk variant in the *APOE* gene cluster. This conclusion is often driven by the results of the conditional association analyses when adjusting the model by, or excluding carriers of, the ɛ4 allele which alters the effects of the other variants in this region (Deelen et al., 2011, 2019; Jun et al., 2012). A recent analysis of haplotypes comprising the rs6857, rs2075650, rs769449, rs429358, and rs7412 SNPs mapped to the *APOE* gene cluster also suggested a nominally significant adverse effect on longevity in carriers of the ɛ3/ɛ3 genotype, that is, independently of the ɛ4 and ɛ2 alleles (Sebastiani et al., 2019).

The ɛ4 allele is well‐known for its strong, adverse association with AD in various populations (Raichlen & Alexander, 2014), which appears to be stronger than its association with longevity and other age‐related diseases (Murabito et al., 2012). Given the impact of the ɛ4 allele in AD research, it has been proposed to reclassify it as a major variant rather than as a risk allele (Genin et al., 2011). Previous studies also argue that the association of the ɛ4 allele with AD risk could be modulated by other variants from the *APOE* gene cluster. Notably, prior research reported the roles of independent and in *cis* combination of long poly thymine repeat polymorphism (rs10524523) and the ε4 allele (Lutz et al., 2016; Roses et al., 2010). Studies of the Central‐Northern Italian population highlighted an adverse role of a haplotype comprising rs405509_T and ε4 alleles in AD risk (Lescai et al., 2011). Furthermore, analyses conditional on the ɛ2 and ɛ4 alleles identified complex haplotypes in an *APOE* cluster comprising 14 or more variants affecting the AD risk independently of the ε4 allele (Zhou et al., 2019).

Here, we examined the chances of living to older ages, that is, 85+ years, for carriers of compound genotypes comprising rs429358 (*APOE* ɛ4 encoding SNP), rs2075650 (*TOMM40*), and rs12721046 (*APOC1*) SNPs. The choice of this triple of SNPs was motivated by previous results showing they contributed to an exceptionally high risk of AD. For example, the risk was 4.37‐fold higher when carriers of the ε4 allele homozygote also carried minor alleles of rs2075650 and rs12721046, compared with carrying major alleles of rs2075650 and rs12721046 (Kulminski et al., 2022).

The results of our analysis yield two main insights. First, we found that the adverse effect of the ɛ4 allele on the chances of living to 85+ years is significantly modulated by minor alleles of the rs2075650 and rs12721046 SNPs. Specifically, carriers of the ɛ4 allele that have minor alleles of rs2075650 and/or rs12721046 were less likely to be found among 85+ year old individuals than carriers of the ɛ4 allele who did not carry the minor alleles of these two SNPs. For example, carrying the ɛ4 allele and heterozygotes of rs2075650 and rs12721046 or two copies of the ɛ4 allele and minor allele homozygotes of rs2075650 and rs12721046 reduced the log odds of living to 85+ years by 26.5% (*β* = −0.265, *p* = 1.48 × 10^−2^) compared with the ɛ4 carriers who did not have minor alleles of either of these SNPs (Figure 1 and Table S3, 111+222).

Second, we showed that after excluding AD subjects, the chances of living to 85+ years for carriers of the ɛ4 allele that have minor alleles of rs2075650 and/or rs12721046 were also consistently smaller in each study and meta‐analysis than the chances for those who did not carry these two minor alleles (Table S4 and Figure 1). Moreover, for the AD‐unaffected subjects, the chances of living to 85+ years were substantially smaller in each study and meta‐analysis than the chances in the samples without consideration of the AD status (Tables S3 and S4, Figure 1). For example, for carriers of the same genotypes as above, the log odds of living to 85+ years were reduced by 1.44 times—from *β* = −0.265 (*p* = 1.48 × 10^−2^) to *β* = −0.382 (*p* = 1.10 × 10^−3^) (Figure 1). Our sensitivity analysis showed that the log odds can be reduced further for living to 87+ years, *β* = −0.504 (*p* = 3.54 × 10^−4^). Given the trend of strengthening of the associations in the AD‐unaffected subjects, excluding subjects who also might be in the preclinical form of AD based on AD biomarkers within the 2018 NIA‐Alzheimer's‐Association framework (Jack et al., 2018; Knopman et al., 2018; Silverberg et al., 2018) may further strengthen the estimates.

The significantly smaller chances of living to 85+ years for AD‐unaffected subjects carrying the ɛ4 allele and minor alleles of rs2075650 and/or rs12721046 SNPs implies the existence of a genetic mechanism that contributes to a shorter life span independently of AD. The substantial (although non‐significant) reduction of these chances in AD‐unaffected subjects suggests a complex interplay of the survival‐ and AD‐related genetic mechanisms. Indeed, it is known that AD affects mortality. For example, a recent meta‐analysis estimated the hazard ratio for mortality in AD patients to be 3.7 (with 1.99–6.88 confidence intervals) (Liang et al., 2021). Our analyses show that carriers of the ɛ4 allele and minor alleles of rs2075650 and/or rs12721046 have substantially smaller chances of living to 85+ years (this work) and have strikingly higher risks of AD (Kulminski et al., 2022) compared with the ɛ4 carriers who do not carry the minor alleles of these two SNPs. A naive expectation is that AD would explain at least a fraction of the association of the identified compound genotypes with survival to 85+ years (i.e., that the magnitude of *β* would be smaller when AD subjects are excluded). Counterintuitive observation of the opposite relationship (i.e., that the magnitude of *β* increases when AD subjects are excluded) supports the complex interplay of the survival‐ and AD‐related mechanisms. This conclusion is in line with prior results showing that neurodegenerative diseases may not modulate the association of the ɛ4 allele with life span (Kulminski et al., 2014). The existence of the survival‐ and AD‐related genetic mechanisms and their potential complex interplay imply genetic heterogeneity, which is an inherent feature of age‐related non‐Mendelian traits (see the Introduction). As to which intermediate phenotypes (endophenotypes) can mediate the associations of the identified compound genotypes with survival to older ages, remains to be elucidated. Given the consistency of the observed associations across different studies regardless of the study design, the endophenotypes should be more general (i.e., beyond the study specific factors), such as other common diseases (e.g., cardiovascular diseases, cancers), ancestry, lifestyle, toxins, and environmental challenges.

Clinical and laboratory studies provide evidence that the *APOE* ε4 allele contributes to AD pathology via AD‐biomarker‐related and biomarker‐independent mechanisms (Yamazaki et al., 2019). The amyloid‐β42 (Aβ42) biomarker of AD was found to be tighter linked with the ε4 allele than AD or mild cognitive impairment (Vemuri et al., 2010). Also, Aβ42 measured in cerebrospinal fluid was independently associated with AD and the ε4 allele (Baek et al., 2020; Lautner et al., 2014). Meanwhile, tau biomarker and neurodegeneration stronger predicted cognitive decline than the ε4 allele (Vemuri et al., 2010). Also, the analysis of positron emission tomography images identified that neurodegeneration is tighter linked to tau pathology than Aβ pathology (Ossenkoppele et al., 2016). These findings suggest that the main contributions of the ε4 allele to AD are through Aβ and tau pathologies. It is hypothesized that the Aβ‐related mechanism is mainly pronounced before symptoms of AD, whereas the AD phenotype develops due to neurodegeneration (Jack et al., 2009; Koutsodendris et al., 2022; Vemuri et al., 2010).

An increasing body of literature reports on the ε4‐dependent mechanisms of AD pathology beyond biomarkers (Yamazaki et al., 2019). For example, ε4 may adversely contribute to AD pathogenesis via a gain of toxic function of pathways linked to insulin signaling and loss of function of the lipid transport pathway, among the other mechanisms. It was also shown that ε4 can affect the function of the blood–brain barrier leading to accelerated cognitive decline (Montagne et al., 2020).

Generally, the shorter lifespan of the ε4 allele carriers is mainly attributed to cardiovascular and neurodegenerative conditions, but the mechanisms are puzzling because the role of the ε4 allele likely changes with age. Indeed, the ε4 allele is ancestral in humans (Fullerton et al., 2000) implying that it was favorable for fitness and survival at reproductive age. However, this allele is minor in all modern human populations, whereas the ε3 allele is major, spreading about 0.266 million years ago (Finch, 2012). This means that the ε4 allele lost a large fraction of its advantage, but not all because it is still retained in humans. A comprehensive review by Trumble and Finch (2019) emphasizes major aspects which underlie the evolutionary advantages of the ε4 allele. Factors contributing to the adaptive advantage of the ε4 allele include cognitive advantage in early life and growth/survival advantages in pathogenic environments. In another review, Finch and Stanford (2004) suggested that adaptation of the new *APOE* alleles was mainly driven by spreading meat consumption. The ε4 allele was less adapted to the consumption of fatty meals, whereas the other alleles favored lower cholesterol concentrations in this environment favoring better cardiovascular health.

The *APOE* cluster harboring *TOMM40*, *APOE*, and *APOC1* genes has a complex regulatory structure with multiple enhancers, which can contribute to regulatory activity in this region affecting cognitive health, lipid metabolism, and immunity (Bekris et al., 2012; Fuior & Gafencu, 2019; Heinemeyer et al., 2019; Lee et al., 2021; Shao et al., 2018). *TOMM40* encodes the essential mitochondrial outer membrane protein (TOM40), which is the main structural component of the channel for delivering proteins into mitochondria. Mitochondria is the main component of immunity (Mills et al., 2017). Interaction of APOE protein regions with mitochondria can contribute to AD pathogenesis via neurotoxicity and mitochondria dysfunction (Chang et al., 2005). Interaction of *TOMM40* with other genes may also modulate lipid metabolism (Zimon et al., 2021). Studies also showed regulatory effects of the *TOMM40* variants, which can affect the expression of both *TOMM40* and *APOE* genes (Linnertz et al., 2014). *APOC1* is a lipid gene modulating lipoprotein metabolism, which directly contributes to cardiovascular health (Fuior & Gafencu, 2019) and can interact with *APOE* affecting the clearance of apolipoproteins (Petit‐Turcotte et al., 2001). *APOC1* variants and the ε4 allele may contribute synergistically to cognitive decline and AD pathogenesis (Zhou et al., 2014).

Thus, prior insights support the view that different mechanisms can drive the chances to live longer lives (e.g., via lipid‐ and immunity‐related mechanisms) and the risk of AD (e.g., via AD‐biomarker‐related mechanisms).

Despite the rigor of our analysis, we acknowledge its limitations. First, the samples used in this analysis were insufficient to robustly examine the role of minor allele homozygotes of rs429358, rs2075650, and rs12721046 SNPs. Second, we did not explore the potential roles of haplotypes comprising these SNPs due to the limited number of minor allele homozygotes. Third, further analyses using relevant studies with potential endophenotypes available are needed to elucidate mediators of the effects between the identified compound genotypes, survival to older ages, and AD risk. Fourth, we did not examine whether the same compound genotypes could be associated with a short life span due to the small number of people who died prematurely. Fifth, we did not examine the potential roles of sex due to the limited sample size.

Thus, this study provides compelling evidence that the adverse effect of the ɛ4 allele on survival to older ages is significantly higher in carriers of minor alleles of rs2075650 and/or rs12721046 SNPs compared with non‐carriers of these alleles. This result is consistent with our prior findings showing that carrying minor alleles of rs2075650 and rs12721046 SNPs significantly increases the AD risk for carriers of the ɛ4 allele. Despite this consistency, our results provide robust evidence that compound genotypes comprising rs429358, rs2075650, and rs12721046 SNPs significantly contribute to the chances to live to older ages in AD‐unaffected subjects. This finding indicates the existence of genetically heterogeneous mechanisms. Prior studies suggest that the chances to live to older ages can be associated with lipid‐ and immunity‐related mechanisms, whereas the AD risk can be driven by the AD‐biomarker‐related mechanism, among others. Targeting heterogeneous contributions of specific combinations of genetic variants to the risk of non‐Mendelian traits helps identify polygenic profiles of individuals at high risk that are promising for the translation of genetic discoveries to health care.

## AUTHOR CONTRIBUTIONS

A.M.K. conceived and designed the experiment and wrote the paper. E.J.W., I.P., and L.H. coded statistical tests, performed statistical analyses. Y.L., E.L., O.B., and K.A. prepared data for the analyses. I.C. performed biological analysis. E.J.W., S.V., A.Y., K.A., E.S., M.F.F., N.S., K.C. and I.C. contributed to writing the paper.

## CONFLICT OF INTEREST

The authors declare no competing interests.

## Supporting information


Appendix S1

## Data Availability

In this article, we used data obtained through dbGaP (accession numbers phs000007.v31 [FHS], phs000287.v7 [CHS], phs000285, v.3 [CARDIA]), the UK Biobank applications #60447 and #62778, and the LLFS study provided by the LLFS Data Management and Coordinating Center (Washington University, St. Louis) and available through dbGaP (accession number phs000397.v3).
