# Supplementary material for: APOE ɛ4 allele and TOMM40‐APOC1 variants jointly contribute to survival to older ages
Source: Aging Cell. 2022 Nov 3;21(12):e13730. doi: 10.1111/acel.13730 (PMC9741507; doi:10.1111/acel.13730)
Supplement: Supplementary file 1 — Appendix S1 [file ACEL-21-e13730-s001.docx]

Supplementary Materials for

***APOE* ɛ4 allele and *TOMM40*-*APOC1* variants jointly contribute to survival to older ages**

Alexander M Kulminski^1*^, Ethan Jain-Washburn^1^, Ian Philipp^1^, Liang He^1^, Yury Loika^1^, Elena Loiko^1^, Olivia Bagley^1^, Svetlana Ukraintseva^1^, Anatoliy Yashin^1^, Konstantin Arbeev^1^, Eric Stallard^1^, Mary F Feitosa^2^, Nicole Schupf^3^, Kaare Christensen^4^, Irina Culminskaya^1^

^1^ Biodemography of Aging Research Unit, Social Science Research Institute, Duke University, Durham, NC, USA.

^2^ Division of Statistical Genomics, Department of Genetics, Washington University School of Medicine, St Louis, MO, USA.

^3^ Gertrude H. Sergievsky Center, Columbia University Irving Medical Center, New York, NY, USA.

^4^ Unit of Epidemiology, Biostatistics and Biodemography, Department of Public Health, Southern Denmark University, Odense, Denmark.

* Corresponding author:

Alexander M. Kulminski

**Address**: Biodemography of Aging Research Unit, Social Science Research Institute, Duke University, Durham, NC 27708, USA

Phone: 919-684-4962; Fax: (919) 684-3861

E-mail: [Alexander.Kulminski@duke.edu](mailto:Alexander.Kulminski@duke.edu)

**This file includes:**

Supplementary Acknowledgement text

Tables S1-S5

**Supplementary Text**

This work was supported by National Institute on Aging (grants R01AG061853, R01AG065477, R01AG070488, U01AG023746, U01AG023712, U01AG023749, U01AG023755, U01AG023744, and U19 AG063893). The funders had no role in study design, data collection, and analysis, decision to publish, or preparation of the manuscript. The content is solely the authors' responsibility and does not necessarily represent the official views of the National Institutes of Health. This article was prepared using a data obtained through dbGaP (accession numbers phs000007.v31 [FHS], phs000287.v7 [CHS], phs000285, v.3 [CARDIA]), the UK Biobank applications #60447 and #62778, and the LLFS study provided by the LLFS Data Management and Coordinating Center (Washington University, St. Louis) and available through dbGaP (accession number phs000397.v3).

**The Framingham Heart Study (FHS)** is a longitudinal multigenerational study on residents of the city of Framingham, Massachusetts. FHS is designed to identify the common factors or characteristics that contribute to cardiovascular disease (CVD) by following its development over a long period of time in a large group of participants who had not yet developed overt symptoms of CVD or suffered a heart attack or stroke. FHS is conducted and supported by the National Heart, Lung, and Blood Institute (NHLBI) in collaboration with Boston University (Contract No. N01-HC-25195 and HHSN268201500001I). This manuscript was not prepared in collaboration with investigators of the FHS and does not necessarily reflect the opinions or views of the FHS, Boston University, or NHLBI. Funding for SHARe Affymetrix genotyping was provided by NHLBI Contract N02-HL-64278. SHARe Illumina genotyping was provided under an agreement between Illumina and Boston University. Funding for CARe genotyping was provided by NHLBI Contract N01-HC-65226. Funding support for the Framingham Dementia dataset was provided by NIH/NIA grant R01 AG08122.

**The Cardiovascular Health Study (CHS)** is a multicenter prospective cohort study of cardiovascular risk factors in ambulatory non-institutionalized men and women aged 65 years and older recruited from four communities: Forsyth County, North Carolina; Sacramento County, California; Washington County, Maryland; and Pittsburgh, Pennsylvania. CHS is designed to determine the importance of conventional CHD risk factors in older adults, and to identify new risk factors in this age group. CHS was supported by contracts HHSN268201200036C, HHSN268200800007C, N01-HC-85079, N01-HC-85080, N01-HC-85081, N01-HC-85082, N01-HC-85083, N01-HC-85084, N01-HC-85085, N01-HC-85086, N01-HC-35129, N01 HC-15103, N01 HC-55222, N01-HC-75150, N01-HC-45133, and N01-HC-85239; grant numbers U01 HL080295 and U01 HL130014 from the National Heart, Lung, and Blood Institute (NHLBI), and R01 AG-023629 from the National Institute on Aging, with additional contribution from the National Institute of Neurological Disorders and Stroke. A full list of principal CHS investigators and institutions can be found at https://chs-nhlbi.org/pi. This manuscript was not prepared in collaboration with CHS investigators and does not necessarily reflect the opinions or views of CHS, or the NHLBI. Additional support for infrastructure was provided by HL105756 and additional genotyping among the African-American cohort was supported in part by HL085251. DNA handling and genotyping at Cedars-Sinai Medical Center was supported in part by National Center for Research Resources grant UL1RR033176, now at the National Center for Advancing Translational Technologies CTSI grant UL1TR000124; in addition to the National Institute of Diabetes and Digestive and Kidney Diseases grant DK063491 to the Southern California Diabetes Endocrinology Research Center.

**UK Biobank (**UKB) is a large-scale biomedical database and research resource, containing in-depth genetic and health information from half a million UK participants. We thank the UK Biobank participants and coordinators for providing this dataset under applications #60447 and #62778.

**The Long Life Family Study** (LLFS) is a longitudinal study designed to determine genetic, behavioral, and environmental factors on participants from families showing exceptional familial longevity. The data are from three field centers in the U.S. and one in Denmark. We thank the LLFS.

**The Coronary Artery Risk Development in Young Adults Study** (CARDIA) is conducted and supported by the National Heart, Lung, and Blood Institute (NHLBI) in collaboration with the University of Alabama at Birmingham (N01-HC95095 & N01-HC48047), University of Minnesota (N01-HC48048), Northwestern University (N01-HC48049), and Kaiser Foundation Research Institute (N01-HC48050). This manuscript was not approved by CARDIA. The opinions and conclusions contained in this publication are solely those of the authors, and are not endorsed by CARDIA or the NHLBI and should not be assumed to reflect the opinions or conclusions of either. Funding for CARe genotyping was provided by NHLBI Contract N01-HC-65226.

**Table S1**. Counts of subjects carrying compound genotypes constructed using rs429358, rs2075650, and rs12721046.

| **MA coding** | **Genotype** | **CHS&UKB** | | | | **FHS** | | | | **LLFS** | | | |
| --- | --- | --- | --- | --- | --- | --- | --- | --- | --- | --- | --- | --- | --- |
|  |  | **Cases** | | **Controls** | | **Cases** | | **Controls** | | **Cases** | | **Controls** | |
|  |  | **N** | **Percent** | **N** | **Percent** | **N** | **Percent** | **N** | **Percent** | **N** | **Percent** | **N** | **Percent** |
| 000 | TT/AA/GG | 1034 | 66.7 | 122360 | 61.1 | 814 | 69.4 | 2441 | 66.0 | 1180 | 73.8 | 533 | 69.2 |
| 001 | TT/AA/Ga | 105 | 6.8 | 10812 | 5.4 | 70 | 6.0 | 189 | 5.1 | 97 | 6.1 | 35 | 4.5 |
| 002 | TT/AA/aa | 0 | 0.0 | 237 | 0.1 | 0 | 0.0 | 3 | 0.1 | 4 | 0.3 | 2 | 0.3 |
| 010 | TT/Ag/GG | 74 | 4.8 | 8507 | 4.2 | 48 | 4.1 | 164 | 4.4 | 78 | 4.9 | 30 | 3.9 |
| 011 | TT/Ag/Ga | 5 | 0.3 | 514 | 0.3 | 20 | 1.7 | 7 | 0.2 | 9 | 0.6 | 6 | 0.8 |
| 012 | TT/Ag/aa | 0 | 0.0 | 6 | 0.0 | 4 | 0.3 | 0 | 0.0 | 1 | 0.1 | 0 | 0.0 |
| 020 | TT/gg/GG | 0 | 0.0 | 161 | 0.1 | 0 | 0.0 | 4 | 0.1 | 2 | 0.1 | 2 | 0.3 |
| 021 | TT/gg/Ga | 0 | 0.0 | 3 | 0.0 | 0 | 0.0 | 0 | 0.0 | 0 | 0.0 | 0 | 0.0 |
| 022 | TT/gg/aa | 0 | 0.0 | 0 | 0.0 | 1 | 0.1 | 1 | 0.0 | 0 | 0.0 | 1 | 0.1 |
| 100 | Tc/AA/GG | 61 | 3.9 | 9013 | 4.5 | 50 | 4.3 | 159 | 4.3 | 50 | 3.1 | 34 | 4.4 |
| 101 | Tc/AA/Ga | 22 | 1.4 | 3080 | 1.5 | 11 | 0.9 | 35 | 0.9 | 8 | 0.5 | 6 | 0.8 |
| 102 | Tc/AA/aa | 1 | 0.1 | 107 | 0.1 | 0 | 0.0 | 2 | 0.1 | 0 | 0.0 | 0 | 0.0 |
| 110 | Tc/Ag/GG | 4 | 0.3 | 1345 | 0.7 | 17 | 1.4 | 20 | 0.5 | 9 | 0.6 | 9 | 1.2 |
| 111 | Tc/Ag/Ga | 212 | 13.7 | 36407 | 18.2 | 116 | 9.9 | 571 | 15.4 | 145 | 9.1 | 105 | 13.6 |
| 112 | Tc/Ag/aa | 12 | 0.8 | 1513 | 0.8 | 8 | 0.7 | 23 | 0.6 | 3 | 0.2 | 0 | 0.0 |
| 120 | Tc/gg/GG | 0 | 0.0 | 36 | 0.0 | 0 | 0.0 | 1 | 0.0 | 0 | 0.0 | 0 | 0.0 |
| 121 | Tc/gg/Ga | 4 | 0.3 | 1248 | 0.6 | 4 | 0.3 | 22 | 0.6 | 5 | 0.3 | 2 | 0.3 |
| 122 | Tc/gg/aa | 0 | 0.0 | 29 | 0.0 | 0 | 0.0 | 0 | 0.0 | 0 | 0.0 | 0 | 0.0 |
| 200 | cc/AA/GG | 2 | 0.1 | 154 | 0.1 | 1 | 0.1 | 1 | 0.0 | 1 | 0.1 | 1 | 0.1 |
| 201 | cc/AA/Ga | 1 | 0.1 | 81 | 0.0 | 0 | 0.0 | 0 | 0.0 | 0 | 0.0 | 0 | 0.0 |
| 202 | cc/AA/aa | 0 | 0.0 | 12 | 0.0 | 0 | 0.0 | 1 | 0.0 | 0 | 0.0 | 0 | 0.0 |
| 210 | cc/Ag/GG | 0 | 0.0 | 33 | 0.0 | 0 | 0.0 | 0 | 0.0 | 0 | 0.0 | 0 | 0.0 |
| 211 | cc/Ag/Ga | 3 | 0.2 | 1291 | 0.6 | 0 | 0.0 | 19 | 0.5 | 5 | 0.3 | 1 | 0.1 |
| 212 | cc/Ag/aa | 0 | 0.0 | 401 | 0.2 | 2 | 0.2 | 4 | 0.1 | 0 | 0.0 | 1 | 0.1 |
| 220 | cc/gg/GG | 0 | 0.0 | 0 | 0.0 | 0 | 0.0 | 0 | 0.0 | 0 | 0.0 | 0 | 0.0 |
| 221 | cc/gg/Ga | 0 | 0.0 | 194 | 0.1 | 1 | 0.1 | 1 | 0.0 | 0 | 0.0 | 0 | 0.0 |
| 222 | cc/gg/aa | 10 | 0.6 | 2648 | 1.3 | 6 | 0.5 | 33 | 0.9 | 3 | 0.2 | 2 | 0.3 |
| **Total** |  | **1550** | **100.0** | **200192** | **100.0** | **1173** | **100.0** | **3701** | **100.0** | **1600** | **100.0** | **770** | **100.0** |

Column "**MA coding**" shows the number of minor alleles in each SNP ordered as rs429358, rs2075650, and rs12721046.

Column "**Genotype**" refers to the actual genotypes of rs429358, rs2075650 and rs12721046, in that order, e.g., TT/Ag/aa refers to the triple of rs429358_TT, rs2075650_Ag, and rs12721046_aa. Upper/lower case denotes here major/minor allele.

UKB = UK Biobank, CHS = Cardiovascular Health Study, FHS = Framingham Heart Study, LLFS = Long-Life Family Study.

Cases are defined as subjects who were 85 years and older; controls are defined as subjects who were younger than 65 years. Because CHS did not include subjects younger than 65 years and UKB did not have subjects 85 years and older, we used pooled sample of CHS and UKB (CHS&UKB).

**Table S2**. The estimates of the log odds *β* of living to 85 years and older for carriers of individual and aggregated compound genotypes comprising rs429358, rs2075650, and rs12721046.

| **Dataset** | **MA**  **coding** | **Genotype** | **Beta** | **SE** | ***p*-value** | **N_case** | **N_control** | **I^2^, %** | ***p*-het** |
| --- | --- | --- | --- | --- | --- | --- | --- | --- | --- |
| CHS&UKB | 000 | TT/AA/GG | Reference | | | 1034 | 122360 |  |  |
| CHS&UKB | 0XY | aggregated | 0.073 | 0.080 | 3.63E-01 | 184 | 20240 |  |  |
| CHS&UKB | 100 | Tc/AA/GG | -0.222 | 0.132 | 9.31E-02 | 61 | 9013 |  |  |
| CHS&UKB | 111 | Tc/Ag/Ga | -0.372 | 0.076 | 8.48E-07 | 212 | 36407 |  |  |
| CHS&UKB | 1XY | aggregated | -0.372 | 0.070 | 1.15E-07 | 255 | 43765 |  |  |
| CHS&UKB | 100+200 | aggregated | -0.207 | 0.130 | 1.12E-01 | 63 | 9167 |  |  |
| CHS&UKB | 111+222 | aggregated | -0.397 | 0.074 | 9.09E-08 | 222 | 39055 |  |  |
| CHS&UKB | 1XY+2XY | aggregated | -0.420 | 0.069 | 9.93E-10 | 269 | 48425 |  |  |
| FHS | 000 | TT/AA/GG | Reference | | | 814 | 2441 |  |  |
| FHS | 0XY | aggregated | 0.135 | 0.117 | 2.45E-01 | 143 | 368 |  |  |
| FHS | 100 | Tc/AA/GG | -0.045 | 0.181 | 8.06E-01 | 50 | 159 |  |  |
| FHS | 111 | Tc/Ag/Ga | -0.517 | 0.118 | 1.27E-05 | 116 | 571 |  |  |
| FHS | 1XY | aggregated | -0.389 | 0.106 | 2.34E-04 | 156 | 674 |  |  |
| FHS | 100+200 | aggregated | -0.029 | 0.180 | 8.73E-01 | 51 | 160 |  |  |
| FHS | 111+222 | aggregated | -0.535 | 0.116 | 3.91E-06 | 122 | 604 |  |  |
| FHS | 1XY+2XY | aggregated | -0.422 | 0.103 | 4.31E-05 | 165 | 732 |  |  |
| LLFS | 000 | TT/AA/GG | Reference | | | 1180 | 533 |  |  |
| LLFS | 0XY | aggregated | 0.026 | 0.163 | 8.72E-01 | 191 | 76 |  |  |
| LLFS | 100 | Tc/AA/GG | -0.469 | 0.256 | 6.66E-02 | 50 | 34 |  |  |
| LLFS | 111 | Tc/Ag/Ga | -0.518 | 0.155 | 8.23E-04 | 145 | 105 |  |  |
| LLFS | 1XY | aggregated | -0.497 | 0.146 | 6.42E-04 | 170 | 122 |  |  |
| LLFS | 100+200 | aggregated | -0.483 | 0.251 | 5.43E-02 | 51 | 35 |  |  |
| LLFS | 111+222 | aggregated | -0.514 | 0.153 | 8.01E-04 | 148 | 107 |  |  |
| LLFS | 1XY+2XY | aggregated | -0.485 | 0.144 | 7.37E-04 | 178 | 126 |  |  |
| ***Meta*** | ***000*** | ***TT/AA/GG*** | ***Reference*** | | | ***3028*** | ***125334*** |  |  |
| ***Meta*** | ***0XY*** | ***aggregated*** | ***0.084*** | ***0.061*** | ***1.72E-01*** | ***518*** | ***20684*** | ***0*** | ***8.45E-1*** |
| ***Meta*** | ***100*** | ***Tc/AA/GG*** | ***-0.206*** | ***0.099*** | ***3.70E-02*** | ***161*** | ***9206*** | ***0*** | ***3.94E-1*** |
| ***Meta*** | ***111*** | ***Tc/Ag/Ga*** | ***-0.429*** | ***0.059*** | ***3.25E-13*** | ***473*** | ***37083*** | ***0*** | ***4.87E-1*** |
| ***Meta*** | ***1XY*** | ***aggregated*** | ***-0.394*** | ***0.054*** | ***3.94E-13*** | ***581*** | ***44561*** | ***0*** | ***7.39E-1*** |
| ***Meta*** | ***100+200*** | ***aggregated*** | ***-0.196*** | ***0.097*** | ***4.36E-02*** | ***165*** | ***9362*** | ***8.30*** | ***3.36E-1*** |
| ***Meta*** | ***111+222*** | ***aggregated*** | ***-0.448*** | ***0.058*** | ***1.01E-14*** | ***492*** | ***39766*** | ***0*** | ***5.42E-1*** |
| ***Meta*** | ***1XY+2XY*** | ***aggregated*** | ***-0.429*** | ***0.053*** | ***6.48E-16*** | ***612*** | ***49283*** | ***0*** | ***9.15E-1*** |

Column "**MA coding**" shows the number of minor alleles in each SNP ordered as rs429358, rs2075650, and rs12721046. Symbols “X” and “Y” denote aggregated compound genotypes; these symbols take values of 0, 1, or 2 but not simultaneously 0.

Column "**Genotype**" refers to the actual genotypes of rs429358, rs2075650 and rs12721046, in that order, e.g., TT/Ag/aa refers to the triple of rs429358_TT, rs2075650_Ag, and rs12721046_aa. Upper/lower case denotes here major/minor allele.

UKB = UK Biobank, CHS = Cardiovascular Health Study, FHS = Framingham Heart Study, LLFS = Long-Life Family Study.

Cases and controls were defined as subjects who were 85 years and older (85+ years) and younger than 65 years at the end of follow-up or right censoring. Because CHS did not include subjects younger than 65 years and UKB did not have subjects 85 years and older, we used pooled sample of CHS and UKB (CHS&UKB).

Meta = Meta-analysis of the results from CHS&UKB, FHS, and LLFS.

I^2^ is the heterogeneity coefficient; *p*-het is the heterogeneity *p*-value.

SE = standard error.

**Table S3**. The reductions of the log odds *β* of living to 85 years and older for carriers of individual and aggregated compound genotypes comprising rs429358, rs2075650, and rs12721046.

| **Dataset** | **MA coding** | **Genotype** | **Beta** | **SE** | ***p*-value** | **N_case** | **N_control** | **I^2^, %** | ***p*-het** |
| --- | --- | --- | --- | --- | --- | --- | --- | --- | --- |
| **Compound genotypes with one ɛ4 allele** | | | | | | | | | |
| CHS&UKB | 100 | Tc/AA/GG | Reference | | | 61 | 9013 |  |  |
| CHS&UKB | 111 | Tc/Ag/Ga | -0.150 | 0.146 | 3.02E-01 | 212 | 36407 |  |  |
| CHS&UKB | 1XY | aggregated | -0.150 | 0.143 | 2.95E-01 | 255 | 43765 |  |  |
| FHS | 100 | Tc/AA/GG | Reference | | | 50 | 159 |  |  |
| FHS | 111 | Tc/Ag/Ga | -0.441 | 0.192 | 2.18E-02 | 116 | 571 |  |  |
| FHS | 1XY | aggregated | -0.314 | 0.189 | 9.60E-02 | 156 | 674 |  |  |
| LLFS | 100 | Tc/AA/GG | Reference | | | 50 | 34 |  |  |
| LLFS | 111 | Tc/Ag/Ga | -0.132 | 0.365 | 7.18E-01 | 145 | 105 |  |  |
| LLFS | 1XY | aggregated | -0.147 | 0.371 | 6.92E-01 | 170 | 122 |  |  |
| ***Meta*** | ***100*** | ***Tc/AA/GG*** | ***Reference*** | | | ***161*** | ***9206*** |  |  |
| ***Meta*** | ***111*** | ***Tc/Ag/Ga*** | ***-0.245*** | ***0.111*** | ***2.69E-02*** | ***473*** | ***37083*** | ***0*** | ***4.59E-1*** |
| ***Meta*** | ***1XY*** | ***aggregated*** | ***-0.204*** | ***0.109*** | ***6.06E-02*** | ***581*** | ***44561*** | ***0*** | ***7.76E-1*** |
|  | | | | | | | | | |
| **Aggregated compound genotypes with one or two ɛ4 alleles** | | | | | | | | | |
| CHS&UKB | 100+200 | aggregated | Reference | | | 63 | 9167 |  |  |
| CHS&UKB | 111+222 | aggregated | -0.190 | 0.143 | 1.85E-01 | 222 | 39055 |  |  |
| CHS&UKB | 1XY+2XY | aggregated | -0.213 | 0.140 | 1.29E-01 | 269 | 48425 |  |  |
| FHS | 100+200 | aggregated | Reference | | | 51 | 160 |  |  |
| FHS | 111+222 | aggregated | -0.459 | 0.190 | 1.55E-02 | 122 | 604 |  |  |
| FHS | 1XY+2XY | aggregated | -0.348 | 0.183 | 5.71E-02 | 165 | 732 |  |  |
| LLFS | 100+200 | aggregated | Reference | | | 51 | 35 |  |  |
| LLFS | 111+222 | aggregated | -0.054 | 0.346 | 8.75E-01 | 148 | 107 |  |  |
| LLFS | 1XY+2XY | aggregated | -0.044 | 0.347 | 8.98E-01 | 178 | 126 |  |  |
| ***Meta*** | ***100+200*** | ***aggregated*** | ***Reference*** | | | ***165*** | ***9362*** |  |  |
| ***Meta*** | ***111+222*** | ***aggregated*** | ***-0.265*** | ***0.109*** | ***1.48E-02*** | ***492*** | ***39766*** | ***0*** | ***4.29E-1*** |
| ***Meta*** | ***1XY+2XY*** | ***aggregated*** | ***-0.243*** | ***0.106*** | ***2.22E-02*** | ***612*** | ***49283*** | ***0*** | ***7.03E-1*** |

Column "**MA coding**" shows the number of minor alleles in each SNP ordered as rs429358, rs2075650, and rs12721046. Symbols “X” and “Y” denote aggregated compound genotypes; these symbols take values of 0, 1, or 2 but not simultaneously 0.

Column "**Genotype**" refers to the actual genotypes of rs429358, rs2075650 and rs12721046, in that order, e.g., TT/Ag/aa refers to the triple of rs429358_TT, rs2075650_Ag, and rs12721046_aa. Upper/lower case denotes here major/minor allele.

UKB = UK Biobank, CHS = Cardiovascular Health Study, FHS = Framingham Heart Study, LLFS = Long-Life Family Study.

Cases are defined as subjects 85 years and older with *APOE* ɛ4; controls are defined as subjects younger than 65 years with *APOE* ɛ4. Because CHS did not include subjects younger than 65 years and UKB did not have subjects 85 years and older, we used pooled sample of CHS and UKB (CHS&UKB).

Meta = Meta-analysis of the results from CHS&UKB, FHS, and LLFS. SE = standard error.

I^2^ is the heterogeneity coefficient; *p*-het is the heterogeneity *p*-value.

**Table S4**. The reductions of the log odds *β* of living to 85 years and older for Alzheimer's disease unaffected carriers of individual and aggregated compound genotypes comprising rs429358, rs2075650, and rs12721046.

| **Dataset** | **MA coding** | **Genotype** | **Beta** | **SE** | ***p*-value** | **N_case** | **N_control** | **I^2^, %** | ***p*-het** |
| --- | --- | --- | --- | --- | --- | --- | --- | --- | --- |
| **Compound genotypes with one ɛ4 allele** | | | | | | | | | |
| CHS&UKB | 100 | Tc/AA/GG | Reference | | | 56 | 9008 |  |  |
| CHS&UKB | 111 | Tc/Ag/Ga | -0.251 | 0.154 | 1.03E-01 | 176 | 36391 |  |  |
| CHS&UKB | 1XY | aggregated | -0.244 | 0.151 | 1.05E-01 | 213 | 43747 |  |  |
| FHS | 100 | Tc/AA/GG | Reference | | | 40 | 159 |  |  |
| FHS | 111 | Tc/Ag/Ga | -0.612 | 0.214 | 4.30E-03 | 78 | 571 |  |  |
| FHS | 1XY | aggregated | -0.473 | 0.217 | 2.89E-02 | 107 | 673 |  |  |
| LLFS | 100 | Tc/AA/GG | Reference | | | 42 | 34 |  |  |
| LLFS | 111 | Tc/Ag/Ga | -0.256 | 0.383 | 5.04E-01 | 110 | 104 |  |  |
| LLFS | 1XY | aggregated | -0.284 | 0.395 | 4.71E-01 | 131 | 121 |  |  |
| ***Meta*** | ***100*** | ***Tc/AA/GG*** | ***Reference*** | | | ***138*** | ***9201*** |  |  |
| ***Meta*** | ***111*** | ***Tc/Ag/Ga*** | ***-0.362*** | ***0.119*** | ***2.28E-03*** | ***364*** | ***37066*** | ***0*** | ***3.76E-1*** |
| ***Meta*** | ***1XY*** | ***aggregated*** | ***-0.316*** | ***0.118*** | ***7.43E-03*** | ***451*** | ***44541*** | ***0*** | ***6.84E-1*** |
|  | | | | | | | | | |
| **Aggregated compound genotypes with one or two ɛ4 alleles** | | | | | | | | | |
| CHS&UKB | 100+200 | aggregated | Reference | | | 57 | 9162 |  |  |
| CHS&UKB | 111+222 | aggregated | -0.272 | 0.152 | 7.31E-02 | 185 | 39038 |  |  |
| CHS&UKB | 1XY+2XY | aggregated | -0.292 | 0.149 | 4.99E-02 | 225 | 48401 |  |  |
| FHS | 100+200 | aggregated | Reference | | | 41 | 160 |  |  |
| FHS | 111+222 | aggregated | -0.660 | 0.212 | 1.82E-03 | 80 | 604 |  |  |
| FHS | 1XY+2XY | aggregated | -0.527 | 0.212 | 1.28E-02 | 112 | 731 |  |  |
| LLFS | 100+200 | aggregated | Reference | | | 42 | 35 |  |  |
| LLFS | 111+222 | aggregated | -0.186 | 0.365 | 6.10E-01 | 111 | 106 |  |  |
| LLFS | 1XY+2XY | aggregated | -0.187 | 0.370 | 6.13E-01 | 135 | 125 |  |  |
| ***Meta*** | ***100+200*** | ***aggregated*** | ***Reference*** | | | ***140*** | ***9357*** |  |  |
| ***Meta*** | ***111+222*** | ***aggregated*** | ***-0.382*** | ***0.117*** | ***1.10E-03*** | ***376*** | ***39748*** | ***21.1*** | ***2.82E-1*** |
| ***Meta*** | ***1XY+2XY*** | ***aggregated*** | ***-0.352*** | ***0.116*** | ***2.35E-03*** | ***472*** | ***49257*** | ***0*** | ***5.92E-1*** |

Column "**MA coding**" shows the number of minor alleles in each SNP ordered as rs429358, rs2075650, and rs12721046. Symbols “X” and “Y” denote aggregated compound genotypes; these symbols take values of 0, 1, or 2 but not simultaneously 0.

Column "**Genotype**" refers to the actual genotypes of rs429358, rs2075650 and rs12721046, in that order, e.g., TT/Ag/aa refers to the triple of rs429358_TT, rs2075650_Ag, and rs12721046_aa. Upper/lower case denotes here major/minor allele.

UKB = UK Biobank, CHS = Cardiovascular Health Study, FHS = Framingham Heart Study, LLFS = Long-Life Family Study.

Cases are defined as subjects 85 years and older with *APOE* ɛ4 but without AD; controls are defined as subjects younger than 65 years with *APOE* ɛ4 but without AD. Because CHS did not include subjects younger than 65 years and UKB did not have subjects 85 years and older, we used pooled sample of CHS and UKB (CHS&UKB).

Meta = Meta-analysis of the results from CHS&UKB, FHS, and LLFS. SE = standard error.

I^2^ is the heterogeneity coefficient; *p*-het is the heterogeneity *p*-value.

**Table S5**. The reductions of the log odds *β* of living to 85 years and older for Alzheimer's disease unaffected carriers of individual and aggregated compound genotypes comprising rs429358, rs2075650, and rs12721046, who do not have ɛ2 allele.

| **Dataset** | **MA coding** | **Genotype** | **Beta** | **SE** | ***p*-value** | **N_case** | **N_control** | **I^2^, %** | ***p*-het** |
| --- | --- | --- | --- | --- | --- | --- | --- | --- | --- |
| **Compound genotypes with one ɛ4 allele** | | | | | | | | | |
| CHS&UKB | 100 | Tc/AA/GG | Reference | | | 48 | 8030 |  |  |
| CHS&UKB | 111 | Tc/Ag/Ga | -0.205 | 0.165 | 2.14E-01 | 159 | 32654 |  |  |
| CHS&UKB | 1XY | aggregated | -0.200 | 0.162 | 2.17E-01 | 194 | 39623 |  |  |
| FHS | 100 | Tc/AA/GG | Reference | | | 36 | 139 |  |  |
| FHS | 111 | Tc/Ag/Ga | -0.643 | 0.226 | 4.50E-03 | 70 | 515 |  |  |
| FHS | 1XY | aggregated | -0.490 | 0.222 | 2.75E-02 | 98 | 614 |  |  |
| LLFS | 100 | Tc/AA/GG | Reference | | | 34 | 28 |  |  |
| LLFS | 111 | Tc/Ag/Ga | -0.411 | 0.468 | 3.80E-01 | 94 | 93 |  |  |
| LLFS | 1XY | aggregated | -0.341 | 0.465 | 4.64E-01 | 113 | 106 |  |  |
| ***Meta*** | ***100*** | ***Tc/AA/GG*** | ***Reference*** | | | ***118*** | ***8196*** |  |  |
| ***Meta*** | ***111*** | ***Tc/Ag/Ga*** | ***-0.361*** | ***0.128*** | ***4.86E-03*** | ***323*** | ***33262*** | ***18.5*** | ***2.93E-1*** |
| ***Meta*** | ***1XY*** | ***aggregated*** | ***-0.303*** | ***0.126*** | ***1.60E-02*** | ***405*** | ***40343*** | ***0*** | ***5.71E-1*** |
|  | | | | | | | | | |
| **Aggregated compound genotypes with one or two ɛ4 alleles** | | | | | | | | | |
| CHS&UKB | 100+200 | aggregated | Reference | | | 49 | 8183 |  |  |
| CHS&UKB | 111+222 | aggregated | -0.230 | 0.163 | 1.58E-01 | 168 | 35298 |  |  |
| CHS&UKB | 1XY+2XY | aggregated | -0.252 | 0.159 | 1.14E-01 | 206 | 44269 |  |  |
| FHS | 100+200 | aggregated | Reference | | | 36 | 139 |  |  |
| FHS | 111+222 | aggregated | -0.696 | 0.224 | 1.84E-03 | 72 | 548 |  |  |
| FHS | 1XY+2XY | aggregated | -0.546 | 0.216 | 1.16E-02 | 103 | 672 |  |  |
| LLFS | 100+200 | aggregated | Reference | | | 34 | 28 |  |  |
| LLFS | 111+222 | aggregated | -0.300 | 0.439 | 4.94E-01 | 95 | 95 |  |  |
| LLFS | 1XY+2XY | aggregated | -0.237 | 0.436 | 5.86E-01 | 117 | 109 |  |  |
| ***Meta*** | ***100+200*** | ***aggregated*** | ***Reference*** | | | ***120*** | ***8351*** |  |  |
| ***Meta*** | ***111+222*** | ***aggregated*** | ***-0.384*** | ***0.126*** | ***2.32E-03*** | ***335*** | ***35941*** | ***30.7*** | ***2.36E-1*** |
| ***Meta*** | ***1XY+2XY*** | ***aggregated*** | ***-0.346*** | ***0.123*** | ***4.91E-03*** | ***426*** | ***45050*** | ***0*** | ***5.31E-1*** |

Column "**MA coding**" shows the number of minor alleles in each SNP ordered as rs429358, rs2075650, and rs12721046. Symbols “X” and “Y” denote aggregated compound genotypes; these symbols take values of 0, 1, or 2 but not simultaneously 0.

Column "**Genotype**" refers to the actual genotypes of rs429358, rs2075650 and rs12721046, in that order, e.g., TT/Ag/aa refers to the triple of rs429358_TT, rs2075650_Ag, and rs12721046_aa. Upper/lower case denotes here major/minor allele

UKB = UK Biobank, CHS = Cardiovascular Health Study, FHS = Framingham Heart Study, LLFS = Long-Life Family Study.

Cases are defined as subjects 85 years and older with *APOE* ɛ4, but not *APOE* ɛ2, and without AD; controls are defined as subjects younger than 65 years with *APOE* ɛ4, but not *APOE* ɛ2, and without AD. Because CHS did not include subjects younger than 65 years and UKB did not have subjects 85 years and older, we used pooled sample of CHS and UKB (CHS&UKB).

Meta = Meta-analysis of the results from CHS&UKB, FHS, and LLFS. SE = standard error.

I^2^ is the heterogeneity coefficient; *p*-het is the heterogeneity *p*-value.

**Table S6**. Sensitivity analysis of the reductions of the log odds *β* of living to older ages for Alzheimer's disease unaffected carriers of aggregated compound genotypes comprising rs429358, rs2075650, and rs12721046.

| **Dataset** | **MA coding** | **Beta** | **SE** | ***p*-value** | **N_case** | **N_control** | **I^2^, %** | ***p*-het** |
| --- | --- | --- | --- | --- | --- | --- | --- | --- |
| **Living to 83 years and older** | | | | | | | | |
| CHS&UKB | 100+200 | Reference | | | 79 | 9162 |  |  |
| CHS&UKB | 111+222 | -0.213 | 0.128 | 9.65E-02 | 272 | 39038 |  |  |
| CHS&UKB | 1XY+2XY | -0.223 | 0.126 | 7.57E-02 | 334 | 48401 |  |  |
| FHS | 100+200 | Reference | | | 53 | 160 |  |  |
| FHS | 111+222 | -0.615 | 0.190 | 1.19E-03 | 108 | 604 |  |  |
| FHS | 1XY+2XY | -0.509 | 0.188 | 6.88E-03 | 147 | 731 |  |  |
| LLFS | 100+200 | Reference | | | 47 | 35 |  |  |
| LLFS | 111+222 | -0.239 | 0.348 | 4.92E-01 | 120 | 106 |  |  |
| LLFS | 1XY+2XY | -0.228 | 0.352 | 5.17E-01 | 148 | 125 |  |  |
| ***Meta*** | ***100+200*** | ***Reference*** | | | ***179*** | ***9357*** |  |  |
| ***Meta*** | ***111+222*** | ***-0.331*** | ***0.102*** | ***1.14E-03*** | ***500*** | ***39748*** | ***36.6*** | ***2.07E-1*** |
| ***Meta*** | ***1XY+2XY*** | ***-0.305*** | ***0.100*** | ***2.38E-03*** | ***451*** | ***44541*** | ***0*** | ***4.39E-1*** |
|  | | | | | | | | |
| **Living to 87 years and older** | | | | | | | | |
| CHS&UKB | 100+200 | Reference | | | 39 | 9162 |  |  |
| CHS&UKB | 111+222 | -0.343 | 0.185 | 6.42E-02 | 118 | 39038 |  |  |
| CHS&UKB | 1XY+2XY | -0.359 | 0.181 | 4.75E-02 | 144 | 48401 |  |  |
| FHS | 100+200 | Reference | | | 33 | 160 |  |  |
| FHS | 111+222 | -1.008 | 0.266 | 1.55E-04 | 48 | 604 |  |  |
| FHS | 1XY+2XY | -0.813 | 0.249 | 1.08E-03 | 70 | 731 |  |  |
| LLFS | 100+200 | Reference | | | 38 | 35 |  |  |
| LLFS | 111+222 | -0.160 | 0.382 | 6.76E-01 | 103 | 106 |  |  |
| LLFS | 1XY+2XY | -0.201 | 0.390 | 6.06E-01 | 123 | 125 |  |  |
| ***Meta*** | ***100+200*** | ***Reference*** | | | ***110*** | ***9357*** |  |  |
| ***Meta*** | ***111+222*** | ***-0.504*** | ***0.141*** | ***3.54E-04*** | ***269*** | ***39748*** | ***61.2*** | ***7.62E-2*** |
| ***Meta*** | ***1XY+2XY*** | ***-0.477*** | ***0.137*** | ***4.98E-04*** | ***337*** | ***49257*** | ***27.3*** | ***2.53E-1*** |

Column "**MA coding**" shows the number of minor alleles in each SNP ordered as rs429358, rs2075650, and rs12721046. Symbols “X” and “Y” denote aggregated compound genotypes; these symbols take values of 0, 1, or 2 but not simultaneously 0.

UKB = UK Biobank, CHS = Cardiovascular Health Study, FHS = Framingham Heart Study, LLFS = Long-Life Family Study.

Cases are defined as subjects 83 or 87 years and older with *APOE* ɛ4 but without AD; controls are defined as subjects younger than 65 years with *APOE* ɛ4 but without AD. Because CHS did not include subjects younger than 65 years and UKB did not have subjects 83 years and older, we used pooled sample of CHS and UKB (CHS&UKB).

Meta = Meta-analysis of the results from CHS&UKB, FHS, and LLFS. SE = standard error.

I^2^ is the heterogeneity coefficient; *p*-het is the heterogeneity *p*-value.

**Table S7**. Sensitivity analysis of the reductions of the log odds *β* of living to 85 years and older for Alzheimer's disease unaffected carriers of individual and aggregated compound genotypes comprising rs429358, rs2075650, and rs12721046 using controls from CARDIA study.

| **Dataset** | **MA coding** | **Genotype** | **Beta** | **SE** | ***p*-value** | **N_case** | **N_control** | **I^2^, %** | ***p*-het** |
| --- | --- | --- | --- | --- | --- | --- | --- | --- | --- |
| **Compound genotypes with one ɛ4 allele** | | | | | | | | | |
| CHS&CARDIA | 100 | Tc/AA/GG | Reference | | | 56 | 75 |  |  |
| CHS&CARDIA | 111 | Tc/Ag/Ga | -0.203 | 0.201 | 3.11E-01 | 176 | 288 |  |  |
| CHS&CARDIA | 1XY | aggregated | -0.217 | 0.197 | 2.70E-01 | 213 | 354 |  |  |
| FHS | 100 | Tc/AA/GG | Reference | | | 40 | 159 |  |  |
| FHS | 111 | Tc/Ag/Ga | -0.612 | 0.214 | 4.30E-03 | 78 | 571 |  |  |
| FHS | 1XY | aggregated | -0.473 | 0.217 | 2.89E-02 | 107 | 673 |  |  |
| LLFS | 100 | Tc/AA/GG | Reference | | | 42 | 34 |  |  |
| LLFS | 111 | Tc/Ag/Ga | -0.256 | 0.383 | 5.04E-01 | 110 | 104 |  |  |
| LLFS | 1XY | aggregated | -0.284 | 0.395 | 4.71E-01 | 131 | 121 |  |  |
| ***Meta*** | ***100*** | ***Tc/AA/GG*** | ***Reference*** | | | ***138*** | ***268*** |  |  |
| ***Meta*** | ***111*** | ***Tc/Ag/Ga*** | ***-0.377*** | ***0.137*** | ***5.91E-03*** | ***364*** | ***963*** | ***2.2*** | ***3.60E-1*** |
| ***Meta*** | ***1XY*** | ***aggregated*** | ***-0.327*** | ***0.137*** | ***1.67E-02*** | ***451*** | ***1148*** | ***0*** | ***6.77E-1*** |
|  | | | | | | | | | |
| **Aggregated compound genotypes with one or two ɛ4 alleles** | | | | | | | | | |
| CHS&CARDIA | 100+200 | aggregated | Reference | | | 57 | 76 |  |  |
| CHS&CARDIA | 111+222 | aggregated | -0.230 | 0.198 | 2.46E-01 | 185 | 310 |  |  |
| CHS&CARDIA | 1XY+2XY | aggregated | -0.250 | 0.194 | 1.98E-01 | 225 | 385 |  |  |
| FHS | 100+200 | aggregated | Reference | | | 41 | 160 |  |  |
| FHS | 111+222 | aggregated | -0.660 | 0.212 | 1.82E-03 | 80 | 604 |  |  |
| FHS | 1XY+2XY | aggregated | -0.527 | 0.212 | 1.28E-02 | 112 | 731 |  |  |
| LLFS | 100+200 | aggregated | Reference | | | 42 | 35 |  |  |
| LLFS | 111+222 | aggregated | -0.186 | 0.365 | 6.10E-01 | 111 | 106 |  |  |
| LLFS | 1XY+2XY | aggregated | -0.187 | 0.370 | 6.13E-01 | 135 | 125 |  |  |
| ***Meta*** | ***100+200*** | ***aggregated*** | ***Reference*** | | | ***140*** | ***271*** |  |  |
| ***Meta*** | ***111+222*** | ***aggregated*** | ***-0.398*** | ***0.135*** | ***3.11E-03*** | ***376*** | ***1020*** | ***22.6*** | ***2.75E-1*** |
| ***Meta*** | ***1XY+2XY*** | ***aggregated*** | ***-0.352*** | ***0.133*** | ***8.37E-03*** | ***472*** | ***1241*** | ***0*** | ***5.61E-1*** |

Column "**MA coding**" shows the number of minor alleles in each SNP ordered as rs429358, rs2075650, and rs12721046. Symbols “X” and “Y” denote aggregated compound genotypes; these symbols take values of 0, 1, or 2 but not simultaneously 0.

Column "**Genotype**" refers to the actual genotypes of rs429358, rs2075650 and rs12721046, in that order, e.g., TT/Ag/aa refers to the triple of rs429358_TT, rs2075650_Ag, and rs12721046_aa. Upper/lower case denotes here major/minor allele.

CARDIA = Coronary Artery Risk Development in Young Adults, CHS = Cardiovascular Health Study, FHS = Framingham Heart Study, LLFS = Long-Life Family Study.

Cases are defined as subjects 85 years and older with *APOE* ɛ4 but without AD; controls are defined as subjects younger than 65 years with *APOE* ɛ4 but without AD. Because CHS did not include subjects younger than 65 years and CARDIA did not have subjects 85 years and older, we used pooled sample of CHS and CARDIA (CHS&CARDIA).

Meta = Meta-analysis of the results from CHS&CARDIA, FHS, and LLFS. SE = standard error.

I^2^ is the heterogeneity coefficient; *p*-het is the heterogeneity *p*-value.
